# Surveillance of vancomycin-resistant enterococci reveals shift in dominating clusters from *vanA* to *vanB Enterococcus faecium* clusters, Denmark, 2015 to 2022

**DOI:** 10.2807/1560-7917.ES.2024.29.23.2300633

**Published:** 2024-06-06

**Authors:** Anette M Hammerum, Kasper Thystrup Karstensen, Louise Roer, Hülya Kaya, Mikkel Lindegaard, Lone Jannok Porsbo, Anne Kjerulf, Mette Pinholt, Barbara Juliane Holzknecht, Peder Worning, Karen Leth Nielsen, Sanne Grønvall Kjær Hansen, Marianne Clausen, Turid S Søndergaard, Esad Dzajic, Claus Østergaard, Mikala Wang, Kristoffer Koch, Henrik Hasman

**Affiliations:** 1 Bacteria, Parasites and Fungi, Statens Serum Institut, Copenhagen, Denmark; 2 Department of Clinical Microbiology, Copenhagen University Hospital - Amager and Hvidovre, Denmark; 3 Department of Clinical Microbiology, Copenhagen University Hospital - Herlev and Gentofte, Herlev, Denmark; 4 Department of Clinical Medicine, University of Copenhagen, Copenhagen, Denmark; 5 Department of Clinical Microbiology, Copenhagen University Hospital, Rigshospitalet, Copenhagen, Denmark; 6 Department of Clinical Microbiology, Odense University Hospital, Odense, Denmark; 7 Department of Clinical Microbiology, Slagelse University Hospital, Slagelse, Denmark; 8 Department of Clinical Microbiology, Sønderjylland Hospital, Aabenraa, Denmark; 9 Department of Clinical Microbiology, Hospital South West Jutland, Esbjerg, Denmark; 10 Department of Clinical Microbiology, Lillebaelt Hospital, Vejle, Denmark; 11 Department of Clinical Microbiology, Aarhus University Hospital, Aarhus, Denmark; 12 Department of Clinical Microbiology, Aalborg University Hospital, Aalborg, Denmark; * These authors contributed equally to this work and share first authorship.

**Keywords:** cgMLST, LRE, LVRE, MLST, *Enterococcus faecalis*, *, Enterococcus faecium*, VRE, WGS

## Abstract

**Background:**

Vancomycin-resistant enterococci (VRE) are increasing in Denmark and Europe. Linezolid and vancomycin-resistant enterococci (LVRE) are of concern, as treatment options are limited. Vancomycin-variable enterococci (VVE) harbour the *vanA* gene complex but are phenotypically vancomycin-susceptible.

**Aim:**

The aim was to describe clonal shifts for VRE and VVE in Denmark between 2015 and 2022 and to investigate genotypic linezolid resistance among the VRE and VVE.

**Methods:**

From 2015 to 2022, 4,090 Danish clinical VRE and VVE isolates were whole genome sequenced. We extracted vancomycin resistance genes and sequence types (STs) from the sequencing data and performed core genome multilocus sequence typing (cgMLST) analysis for *Enterococcus faecium*. All isolates were tested for the presence of mutations or genes encoding linezolid resistance.

**Results:**

In total 99% of the VRE and VVE isolates were *E. faecium.* From 2015 through 2019, 91.1% of the VRE and VVE were *vanA E. faecium*. During 2020, to the number of *vanB E. faecium* increased to 254 of 509 VRE and VVE isolates. Between 2015 and 2022, seven *E. faecium* clusters dominated: ST80-CT14 *vanA*, ST117-CT24 *vanA*, ST203-CT859 *vanA,* ST1421-CT1134 *vanA* (VVE cluster)*,* ST80-CT1064 *vanA/vanB*, ST117-CT36 *vanB* and ST80-CT2406 *vanB.* We detected 35 linezolid vancomycin-resistant *E. faecium* and eight linezolid-resistant VVEfm.

**Conclusion:**

From 2015 to 2022, the numbers of VRE and VVE increased. The spread of the VVE cluster ST1421-CT1134 *vanA E. faecium* in Denmark is a concern, especially since VVE diagnostics are challenging. The finding of LVRE, although in small numbers, ia also a concern, as treatment options are limited.

**Key public health message**

**What did you want to address in this study and why?**

Vancomycin is an antibiotic used to treat serious infections caused by multidrug-resistant bacteria. Vancomycin-resistant enterococci (VRE) are bacteria that can cause infections there are difficult to treat; they have become more frequent in Denmark and Europe in the last decade. Infections with VRE enterococci can be treated with another antibiotic: linezolid. We wanted to investigate how many vancomycin-resistant Enterococci were also resistant to linezolid.

**What have we learnt from this study?**

Detections of VRE in Denmark have increased from 520 isolates in 2015 to 827 isolates in 2022. During those years, the highest number of VRE occurred in the Capital Region of Denmark. From 2019 to 2022, VRE and VVE in this region decreased, but VRE increased in the Zealand Region, Central Denmark Region and North Denmark Region. Only 1% of the vancomycin-resistant enterococci were also resistant to linezolid.

**What are the implications of your findings for public health?**

The increase in VRE is of concern because this can lead to an increase in the use of other antimicrobial agents i.e. linezolid or daptomycin. Moreover, finding VRE that are also resistant to linezolid, even though the numbers are low, is a concern because the treatment options for such infections are very limited.

## Background

*Enterococcus faecalis* and *Enterococcus faecium* can cause urinary tract infections, intra-abdominal infections, skin infections and bloodstream infections [1]. *Enterococcus faecium* belongs to the ESKAPE bacteria, a group of six pathogens which includes *Staphylococcus aureus, Klebsiella pneumoniae, Acinetobacter baumannii*, *Pseudomonas aeruginosa* and *Enterobacter* spp. besides *E. faecium* [2]. They can be difficult to treat since they are intrinsically resistant to many antibiotics such as cephalosporins, trimethoprim-sulfamethoxazole and lincosamides [3]. Furthermore, *E. faecium* can acquire antibiotic resistance through chromosomal mutations or gene exchange. High-level resistance to aminoglycosides and resistance to ampicillin and glycopeptides are frequent in *E. faecium* [4]. Many *E. faecium* are multidrug-resistant, and they are a leading cause of nosocomial infections worldwide [5]. During the last 10 years, vancomycin-resistant *E. faecium* (VREfm) have increased in several countries in Europe [6]. Eight types of acquired vancomycin resistance genes, *vanA, vanB, vanD, vanE, vanG, vanL, vanM* and *vanN*, have been reported [7]. In Europe, *vanA* and *vanB* are most prevalent among clinical VREfm isolates. Vancomycin-variable enterococci (VVE) are *E. faecium* that have the *vanA* gene complex but are phenotypically vancomycin-susceptible [8]. In the laboratory, VVE can only be detected by molecular methods and cannot be cultured on selective vancomycin-containing medium. They are capable of shifting from a vancomycin-susceptible phenotype to a resistant phenotype during vancomycin therapy in patients, thus limiting the success of treatment and representing an important source for vancomycin resistance genes [9].

In general, if VRE are highly resistant to other antimicrobial agents, the two major remaining antibiotics are linezolid and daptomycin [10]. Resistance to linezolid in enterococci is often due to mutations in the V domain of the 23S rRNA gene (G2576T or G2505A). Furthermore, transferable resistance genes encoding linezolid resistance in *Enterococcus* spp., *cfr*, *cfr*(B), *optrA* and *poxtA*, have been described. A web tool, LRE-Finder, has been developed for detection of the 23S rRNA mutations and the optrA, cfr, cfr(B) and poxtA genes encoding linezolid resistance in enterococci from whole-genome sequences [11]. To our knowledge, no web tools have been developed for detection of daptomycin resistance or tigecycline from whole genome sequencing (WGS) data.

We have previously described the surveillance of VRE and VVE in clinical isolates in Denmark from 2005 to first quarter of 2019 [12,13]. In the present study, we describe *van* genes detected in the VRE and VVE isolated from 2015 to 2022. We investigated whether major *E. faecium* clusters were present both at regional and national level. Furthermore, we tested VRE and VVE isolates for mutations and genes encoding linezolid resistance.

## Methods

### Healthcare regions

**Figure 1:**
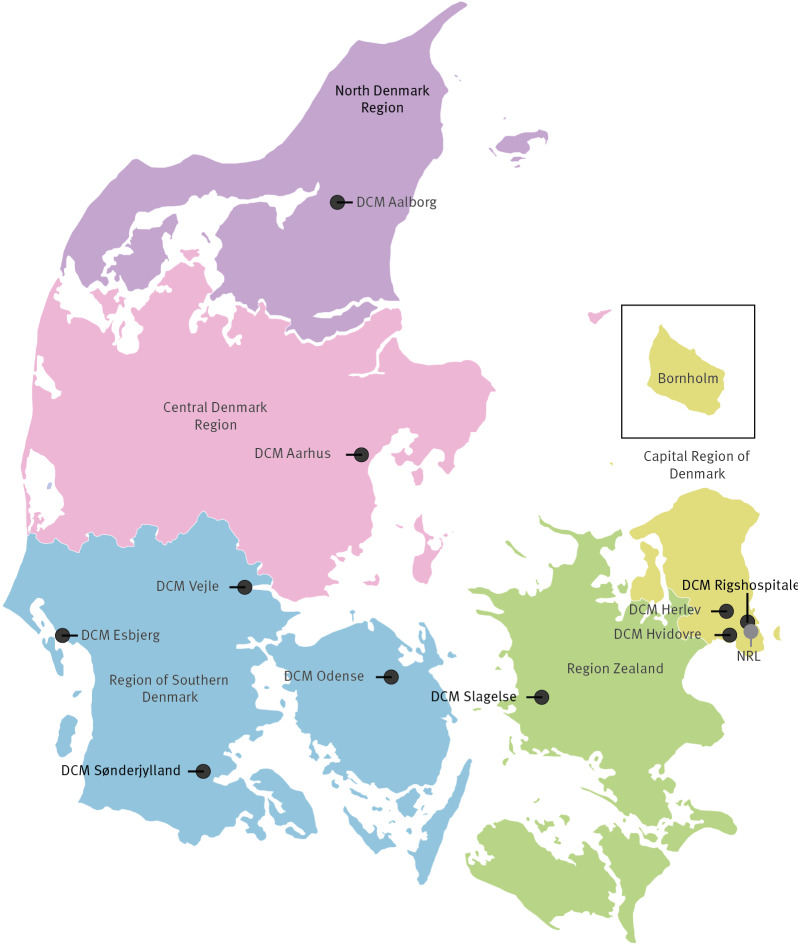
The five healthcare regions and the 10 Departments of Clinical Microbiology and the National Reference Laboratory for Antimicrobial Resistance, Statens Serum Institut, Denmark, 2022

DCM: Department of Clinical Microbiology; NRL: National Reference Laboratory for Antimicrobial Resistance, Statens Serum Institut.

Denmark is divided into five geographical and administrative regions (Figure 1): Capital Region of Denmark (1,867,948 inhabitants, 970,169 bed-days, containing the Department of Clinical Microbiology (DCM) Rigshospitalet, DCM Herlev, DCM Hvidovre), Region Zealand (843,513 inhabitants, 450,438 bed-days, containing DCM Slagelse), Region of Southern Denmark (1,228,362 inhabitants, 606,951 bed-days, containing; DCM Odense, DCM Vejle, DCM Sønderjylland and DCM Esbjerg), Central Denmark Region (1,341,856 inhabitants, 614,411 bed-days, containing DCM Aarhus) and North Denmark Region (246,566 inhabitants, 334,698 bed-days, containing DCM Aalborg) [14].

### Vancomycin-resistant and vancomycin-variable enterococcal isolates

In Denmark, VRE are not notifiable. The 10 DCMs receive samples from hospitals and general practitioners in the five regions. They isolate *Enterococcus* spp. from different clinical samples, e.g. urine, blood and tissue. Vancomycin resistance is detected using a phenotypical method according to EUCAST [15]. Furthermore, VVE are detected by PCR of *E. faecium* isolates. Since 2015, the 10 DCMs have voluntarily submitted to the National Reference Laboratory for Antimicrobial Resistance at Statens Serum Institut (NRL/SSI) the VRE and VVE isolates from clinical samples, but not (faecal) isolates from screening [12]. 

We included in this study only one VRE and VVE isolate per patient within a 12-month period, except if both an *E. faecalis* and an *E. faecium* were detected from the same patient within the same year. The personal identification number was used as identifier, so even when a patient with VRE was moved to another hospital, only one VRE was included. 

To determine any under-reporting in the submissions, we compared the number of VRE and VVE submitted to SSI with data from clinical VRE reported by the DCMs to the Danish Microbiology DataBase (MiBa). The MiBa database serves both as a tool for clinical doctors to access clinical microbiology test results on their patients and a national surveillance and research database [16]. Data from microbiological analyses performed by the DCMs are registered in MiBa. Each test report in MiBa contains information on the individual case (personal identification number, age at sample, sex), the sample (sample ID, sampling date and time, specimen type (blood, urine, etc)), the diagnostic analyses performed, and results including microbiological species, phenotypic antimicrobial susceptibility and in some cases resistance genes (e.g. *vanA* and *vanB*) [17].

### Whole genome sequencing typing

From 2015 through 2022, all clinical VRE and VVE isolates (n = 4,090) underwent WGS. Genomic DNA was extracted (DNeasy Blood and Tissue Kit, Qiagen), with subsequent library construction (Nextera Kit, Illumina), and WGS was performed (Nextseq, Illumina) to obtain paired-end reads of 2 × 150 bp. The quality and quantity of the raw reads of all isolates were assessed using the Bifrost QC pipeline and assembled into draft genomes using the SKESA assembler version 2.2. Quality control, detection of resistance genes and multilocus sequence types (MLST) as well as species identification was carried out using the Bifrost QC pipeline using the MLST and ResFinder databases available at the Center of Genomic Epidemiology homepage (www.cge.food.dtu.dk). The isolates were further subtyped in SeqSphere+ version 8.5.1 (Ridom GmbH) using the *E. faecium* cgMLST scheme by de Been et al. [18]. Local single linkage clustering (SLC) identification numbers were defined for *E. faecium* isolates, clustering isolates with up to 20 allellic distances, sometimes including multiple complex types. The local SLC clusters were named after the complex type of the earliest observed *E. faecium* isolate within each cluster [18].

Furthermore, we tested all VRE and VVE isolates from 2015 to 2022 for the mutations and genes encoding linezolid resistance using the LRE-Finder. The LRE-Finder detects the fraction of thymine bases in position 2,576 and the fraction of adenine bases in position 2,505 of the 23S rRNA and the *cfr, cfr(B), optrA* and *poxtA* genes by aligning raw sequencing reads (fastq format) with k-mer alignment [11].

## Results

### Analysis related to typing

**Figure 2:**
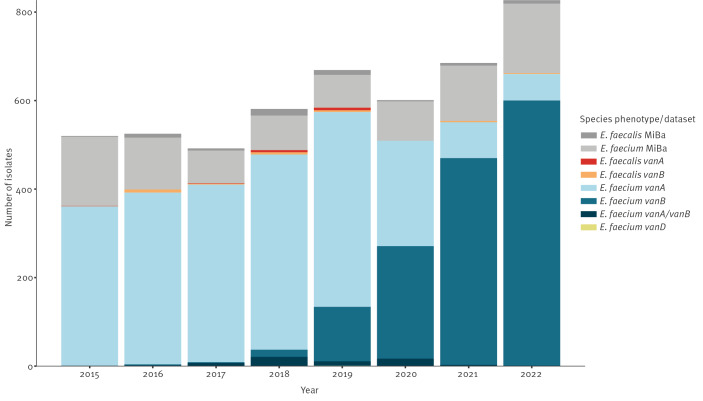
*Enterococcus faecium* and *Enterococcus faecalis* *van* genes per year and isolates, Denmark, 2015–2022 (n = 4,862)

From 2015 to 2022, a total of 4,090 VRE and VVE were sent to SSI. Most of the samples were isolated from urine (65%), 11% were from blood, and the rest were from other clinical specimen (e.g. wounds, tracheal secretions or pus from the abdomen). In total, 2,504 *vanA E. faecium*, 1,485 *vanB E. faecium*, 62 *vanA/vanB E. faecium*, one *vanD E. faecium*, 15 *vanA E. faecalis* and 23 *vanB E. faecalis* from clinical samples were submitted to SSI (Figure 2).

The graph contains isolates submitted to the National Reference Laboratory for Antimicrobial Resistance, Statens Serum Institut (n = 4,090) and unsubmitted isolates from the The Danish Microbiology DataBase (n = 772). The single *vanD E. faecium* was detected in 2019.

We identified additional VRE and VVE isolates in MiBa which had not been submitted to SSI: 59 *E. faecalis* and 713 *E. faecium*. These isolates were not genotyped. They are named *E. faecium* MiBa and *E. faecalis* MiBa in Figure 2. Adding the MiBa isolates to those from the NRL/SSI, a total of 520 VRE and VVE isolates were detected in 2015. This increased to 827 VRE and VVE isolates in 2022 (Figure 2). Until 2020, *vanA E. faecium* were most prevalent. Since 2021, *vanB E. faecium* has dominated (Figure 2). 

**Figure 3:**
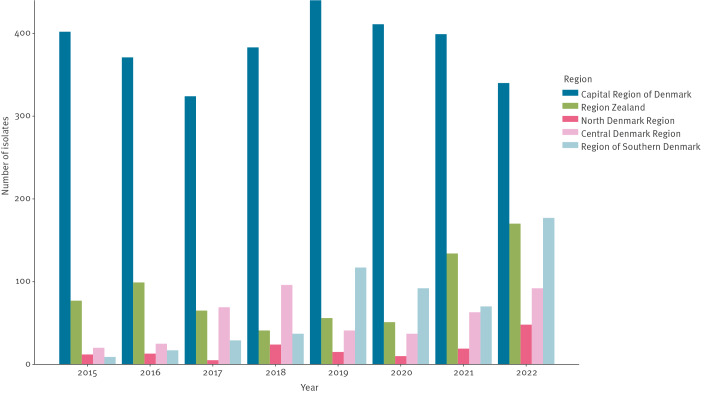
Numbers of vancomycin-resistant and vancomycin-variable enterococci per region per year, Denmark, 2015–2022 (n = 4,862)

Figure 3 shows the number of clinical VRE and VVE for the five Danish regions per year from 2015 to 2022, assigned by testing DCM. The numbers are a combination of data from the NRL/SSI and MiBa. The Capital Region had the highest number of VRE and VVE. From 2019 to 2022, the number of VRE and VVE in the Capital Region decreased. In the same period, VRE increased in the Zealand Region, Central Denmark Region and North Denmark Region.

In Supplementary Figure S1, we additionally provide the incidence of VRE and VVE reported as VRE and VVE per 1,000 bed-days. When looking at VRE and VVE in relation to hospital activity during 2022, the Zealand Region had the highest incidence of VRE and VVE per 1,000 bed-days.

### Evolution of *vanA* and *vanB Enterococcus faecium*

**Figure 4:**
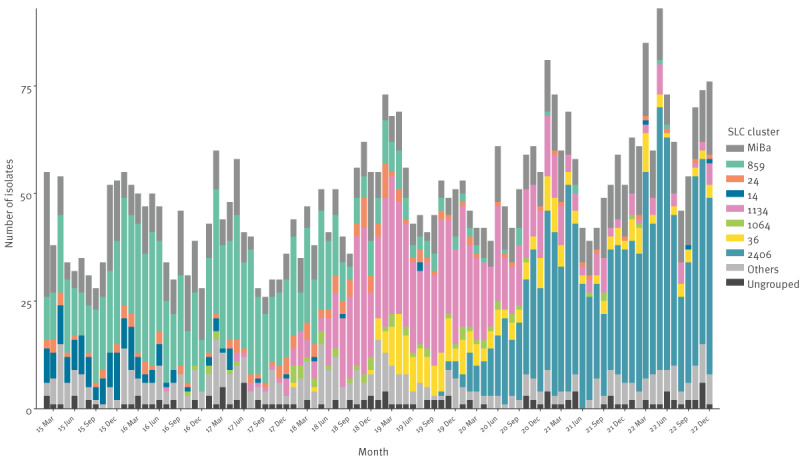
Monthly distribution of the seven most prevalent vancomycin-resistant and vancomycin-variable *Enterococcus faecium* clusters, Denmark, 2015–2022 (n = 4,862)

MiBa: Danish Microbiology DataBase; SLC: single linkage clustering.

**Figure 5:**
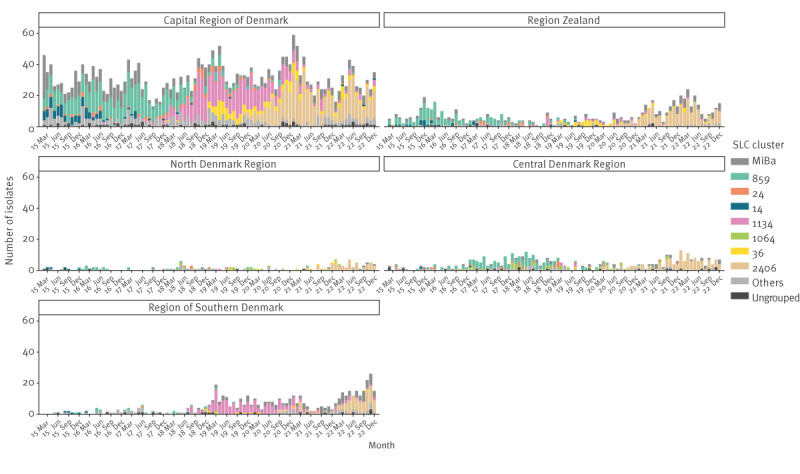
Monthly distribution of the seven most prevalent vancomycin-resistant *Enterococcus faecium* clusters clusters, by region, Denmark, 2015–2022 (n = 4,862)

From 2015 to 2022, seven *E. faecium* clusters dominated: ST80-CT14 *vanA*, ST117-CT24 *vanA*, ST203-CT859 *vanA,* ST1421-CT1134 *vanA,* ST80-CT1064 *vanA/vanB*, ST117-CT36 *vanB* and ST80-CT2406 *vanB.* Figure 4 shows the number of these seven clusters per month during the period 2015 to 2022. In Figure 5, the seven clusters are shown per region during 2015 to 2022. 

The seven most prevalent clusters are included, the numbers in the figure relate to the CT cluster numbers: 14 = ST80-CT14 *vanA*; 24 = ST117-CT24 *vanA*; 859 = ST203-CT859 *vanA;* 1134 = ST1421-CT1134 *vanA;* 1064 = ST80-CT1064 *vanA/vanB*; 36 = ST117-CT36 *vanB* and 2406 = ST80-CT2406 *vanB.*

SLC: single linkage clustering.

The seven most prevalent clusters are included, the numbers in the figure relate to the CT cluster numbers: 14 = ST80-CT14 *vanA*; 24 = ST117-CT24 *vanA*; 859 = ST203-CT859 *vanA;* 1134 = ST1421-CT1134 *vanA;* 1064 = ST80-CT1064 *vanA/vanB*; 36 = ST117-CT36 *vanB* and 2406 = ST80-CT2406 *vanB.*

We detected ST80-CT14 *vanA E. faecium* in all Danish Regions during 2015. On a national level, the numbers of ST80-CT14 *vanA E. faecium* decreased from 2016 to 2018. In 2022, only a few isolates ST80-CT14 *vanA E. faecium* were detected in the Capital Region. 

ST117-CT24 *vanA E. faecium* was first detected in the Capital Region in January 2015. Between 2015 and 2022 it was detected in all five regions.

ST203-CT859 *vanA E. faecium* was first detected in Denmark in December 2014 (data not shown). It was one of the dominating clones from 2015 through July 2018 and was detected in all five regions. In 2022, only a few isolates belonging to ST203-CT859 *vanA E. faecium* were detected.

ST80-CT1064 *vanA-vanB E. faecium* was first detected in Central Denmark Region in October 2016. It spread to the North Jutland Region during April 2018. During 2019, a single case of ST80-CT1064 *vanA-vanB E. faecium* was detected in the Southern Region, but it was not detected in the Capital Region and the Region Zealand. This clone disappeared during January 2022.

*E. faecium* isolates belonging to ST1421-CT1134 *vanA E. faecium* (VVE cluster) were first detected in clinical samples in 2016. In 2018, 34% of the *E. faecium* isolates belonged to ST1421-CT1134, and they were mostly detected in The Capital Region. Testing by *vanA* PCR was first implemented in Region Zealand and Central Denmark Region during 2019. During the period 2019 to 2022, only few ST1421-CT1134 *vanA E. faecium* were detected in Region Zealand, Central Denmark Region and North Denmark Region. During 2019, ST1421-CT1134 *vanA E. faecium* was the most prevalent type (44%) but decreased during 2021 and 2022.

ST117-CT36 *vanB E. faecium* was detected in January 2019 in Capital Region of Denmark. During 2019, ST117-CT36 *vanB E. faecium* was detected in all five Danish Regions, but only in very low numbers in Central Denmark Region and North Denmark Region.

In October 2019, the first clinical ST80-CT2406 *vanB E. faecium* sample was detected in a patient hospitalised in the Capital Region. It spread further to other patients in the Capital Region in 2019. During 2020, this cluster was detected in all Danish regions except the Northern Region of Denmark but during 2021, it was also detected there.

### Linezolid- and vancomycin-resistant enterococci

We investigated WGS data from the VRE and VVE isolates for 23S rRNA mutations and *optrA, cfr, cfr*(B) and *poxtA* genes using the LRE-Finder. From 2015 through 2022, 35 linezolid- and vancomycin-resistant *E. faecium* (LVREfm) and eight linezolid-resistant VVEfm (LVVEfm) were detected, whereas no linezolid- and vancomycin-resistant *E. faecalis* were detected. The LVREfm and LVVEfm were from all five regions except Central Denmark Region. The *vanA* gene was detected in 11 LVREfm and eight LVVEfm isolates, whereas *vanB* was detected in 24 LVREfm isolates. Linezolid resistance was encoded by the G2576T 23S rRNA mutation in the LVVEfm isolates and was also often most often detected among the LVREfm isolates (Table).

**Table:** Genetic characterisation of linezolid- and vancomycin-resistant *Enterococcus faecium* isolates, Denmark 2015–2022 (n =35)

Vancomycin resistance gene	Linezolid resistance mutation and/or gene			
	G2576T^a^	G2576T and *optrA*	*optrA*	*cfrB*
*vanA*	7	1	3	0
*vanB*	23	0	0	1

^a^ The number of isolates which are mutated in position 2,576 of the 23S rRNA.

## Discussion

The numbers of VRE and VVE in Denmark increased from 520 isolates in 2015 to 827 isolates in 2022. In total, 19% of the isolates were not sent to SSI, but detected from MiBa. Those 19% were distributed evenly throughout the study period and it did not seem to be a systematic loss. Since these MiBa isolates not were typed, it is hard to know which *van* genes and cgMLST they had. From 2015 through 2019, 91.1% of the VRE and VVE were *vanA E. faecium*. During 2020, a shift started from *vanA E. faecium* to *vanB E. faecium*. A similar shift was detected in the end of the 2000s in Germany [19]. In 2020, *vanB*-positive *E. faecium* were still dominating in Germany [20]. Also in the Netherlands, a shift occurred from *vanA E. faecium* outbreaks in 1999 to 2014 to *vanB E. faecium* outbreaks between 2014 and 2017 [21]. Until 2019, *vanB E. faecium* were more prevalent than *vanA E. faecium* in Norway [22].

In general, *vanD E. faecium* isolates are rarely reported [19]. In our study, only one *vanD E. faecium* was detected, while *vanD* enterococci have occasionally been detected in Germany, the Netherlands, Norway and Sweden (personal communication; Caroline Kaipe, August 2023) [19,22,23].

The number of VRE differed between the Danish regions, which can partly be explained by different laboratory methods used in different DCMs but also by differences in the type of specimen tested for VRE. One DCM in the Region of Southern Denmark did not test enterococci from urine for vancomycin susceptibility (data not shown). As VRE are most often detected from urine on a national basis, a large number of VRE might be missing from this DCM. As this DCM had overlap in patients with other hospitals in the region, we would expect that the distribution of *van* genes and genetic clusters would be the same as the tested VRE isolates from the rest of the region.

The use of molecular diagnostics to detect VVE influences the detection of VVE. Since the emergence of the VVE cluster ST1421-CT1134, most DCMs have been using *vanA* PCR on all invasive isolates, some also on all clinical isolates. The difference from DCM to DCM in the use of *vanA* PCR for detection of VVE is a bias, but it is hard to know how big this bias is.

There are national guidelines on infection prevention and control measures for VRE, but VRE are not notifiable in Denmark [24]. Therefore, the infection prevention and control measures, such as screening algorithms and isolation precautions, differ in the different regions. In 2023, six of 10 DCMs tested for VRE if the patient had been hopitalised outside the Nordic countries within the last 6 months. Another difference was isolation strategies: eight of 10 DCMs isolated patients with VRE. The local guidelines have changed over time, making it difficult to draw clear conclusions about the interaction between diagnostics, infection prevention and control measures and the changing epidemiology.

The Capital Region had the highest number of VRE and VVE overall, however, the numbers in that region decreased from 2019 to 2022. In general, specific measures to prevent transmission of VRE and VVE were particularly strict in the Capital Region, compared to the other regions. Furthermore, this region uses laboratory algorithms focused on rapid diagnostics (e.g. clone-specific PCRs and *vanA* and *vanB* PCRs on enrichment broth).

The Capital Region also has the highest hospital activity in the country, both in number of bed-days and number of admissions [14]. When looking at VRE and VVE in relation to hospital activity in 2022, the Zealand Region had the highest incidence of VRE and VVE per 1,000 bed-days, which was higher than the VRE and VVE incidence in the Capital Region.

From 2019 to 2022, an increase in VRE was detected for the Zealand Region, Central Denmark Region and North Denmark Region. This a higher workload for hospital staff in charge of patient isolation and outbreak investigations. We did not examine the economic cost associated with VRE infections in healthcare, but an increase in the number of VRE can be expected to have financial implications [25].

Between 2015 and 2022, seven *E. faecium* clusters dominated; ST80-CT14 *vanA*, ST117-CT24 *vanA*, ST203-CT859 *vanA,* ST1421-CT1134 *vanA,* ST80-CT1064 *vanA/vanB*, ST117-CT36 *vanB* and ST80-CT2406 *vanB*. Each of these clusters were present for several years and then disappeared. We here describe each cluster in relation to its spread between the Danish regions, its possible origin and spread to other countries.

During 2012 and 2013, ST80-CT14 *vanA E. faecium* was highly prevalent in the Capital Region. This is similar to the Group2_ST80 in the study by Pinholt et al. analysing Danish data from January 2012 to April 2013 [26]. ST80-CT14 *vanA E. faecium* occurred in all Danish Regions during 2015. To our knowledge, ST80-CT14 *vanA E. faecium* has not been reported outside Denmark, so the origin of this cluster is unknown.

ST117-CT24 *vanA E. faecium* was first detected in the Capital Region in January 2015; its origin is unknown. Since the late 1990s, ST117-CT24 *vanA E. faecium* has spread in several regions in Germany and was also detected in the Netherlands in 2014 [19,27]. Furthermore, an Irish/Danish study detected ST1478-CT24 (a single locus variant of ST117) VREfm from Ireland, with 15 allelic differences to the nearest Danish ST117-CT24 VREfm isolate [28]. It therefore seems likely that the Danish ST117-CT24 VREfm isolate was imported to Denmark from another European country.

Two of the ‘Danish’ clusters, ST1421-CT1134 *vanA E. faecium* and ST203-CT859 *vanA E. faecium,* spread to all Danish regions and other Nordic countries. The ST203-CT859 *vanA E. faecium* cluster was first detected in Denmark in December 2014. The origin of this cluster is unknown. It spread to the south of Sweden and the Faroe Islands during 2015 [12]. In 2020, the ST203-CT859 *vanA E. faecium* cluster was detected in Norway (personal communication: Kristin Hegstad, August 2023).

*Enterococcus faecium* isolates belonging to the ST1421-CT1134 *vanA E. faecium* (VVE clone) were first detected in clinical samples in 2016 in the Capital Region. During 2019, ST1421-CT1134 *vanA E. faecium* was the most prevalent type (44%) [13]. Furthermore, ST1421-CT1134 *vanA E. faecium* spread to the Faroe Islands during 2018 and 2019 [13]. The origin of the VVE clone, ST1421-CT1134 *vanA E. faecium* is unknown, but ST1421-CT1134 *vanA E. faecium* isolates have been detected in Australia, Japan, Korea and Tasmania [29-32].

ST80-CT1064 *vanA-vanB E. faecium* was first detected in Central Denmark Region in October 2016. This cluster was the only one that did not spread to all Danish regions. The origin of this cluster is unknown and VRE that contain both the *vanA* and the *vanB* gene are unusual. Moreover, ST17 *vanA/vanB E. faecium* isolates have been reported in Vietnam and ST117 *vanA/vanB E. faecium* isolates in Greece [33,34].

Two clusters, ST117-CT36 *vanB E. faecium* and ST80-CT2406 *E. faecium,* were introduced via patients transferred from Germany to Danish hopitals. Pinholt et al. describe the emergence and spread of ST117-CT36 *vanB E. faecium* in the Capital Region of Denmark [35]: The index patient with ST117-CT36 *vanB E. faecium* was transferred from Germany to a hospital in the Capital Region of Denmark in January 2019. According to the cgMLST database, the first detection of ST117-CT36 *E. faecium* was in 2015. Weber et al. described ST117-CT36 *vanB E. faecium* as highly prevalent in Germany in 2015 [36]. This shows that the cluster has endemic potential. A clone-specific PCR which can detect ST117-CT36 *vanB E. faecium* was used in the Capital region. One could speculate that this may be the reason why this cluster was not as successful as others, because this particular PCR allowed fast identification and elimination of this cluster. 

The first report of ST80-CT2406 *E. faecium* in the cgMLST *E. faecium* database was during 2019. In August 2019, the first detection of ST80-CT2406 *vanB E. faecium* was a screening sample from a patient transferred from a German hospital before detection (personal communication: Mette Pinholt, August 2023).

We found spread of seven clusters during 8 years. In some cases, the same clusters were seen abroad and in two cases, the high epidemic clusters were imported. A global dissemination of hospital clones of *E. faecium* has also been described [37]. This emphasises the need to test for VRE if the patient had been hospitalised abroad. 

We do not know why these seven *E. faecium* clusters were more successful than other VREfm types. It can be investigated whether these clones have more potential virulence genes that the other VREfm types. This can be done using the updated *E. faecium*/*E. lactis* Virulence-Finder [38]. An Australian VRE study found that major strains of *E. faecium* isolated over 15 years showed unique virulome and resistome profiles with no indication of increasing virulence or antimicrobial resistance determinants. The strains were distantly related and the acquisition of different genes encoding similar antimicrobial resistances suggested the independent evolution of each strain [39].

Our study included only clinical VRE and VVE. Whether this distribution is also representative of colonisation with enterococci is unknown. A small study from DCM Hvidovre, Denmark examined whether a patient with a positive VRE clinical sample had the same VREfm in a preceding screening sample (within 60 days). The VREfm pairs (screening isolate and invasive isolate) were sequenced with WGS. Of 19 VREfm pairs, cgMLST types matched in 13, and one additional pair had matching *vanA* plasmids. Infection was thus preceded by colonisation with the same isolate in 13 of 19 patients [40].

Linezolid is indicated for Gram-positive infections and approved for the treatment of bacterial pneumonia, skin and skin structure infections, and VRE infections, including infections complicated by bacteraemia [41]. In Denmark, the consumption of linezolid has increased from 0.48 defined daily doses (DDD) per 100 bed-days in 2015 to 0.65 DDD per 100 bed-days in 2022 [14]. Using the LRE-Finder, we detected 45 LVREfm isolates among the 4,052 VREfm and VVEfm isolates; this prevalence was low (1.1%). A low prevalence of LVRE is also seen in Germany [42]. Development of linezolid resistance can develop under treatment with linezolid. We did not have data on linezolid consumption for the individual patients in our study. We investigated the first VRE isolate per 12 months, so it seems likely that the patients were treated with linezolid afterwards. A recent Danish study identified six patients who initially carried a vancomycin-resistant, linezolid-sensitive *Enterococcus*, but from whom LVRE closely related to their initial isolate were recovered after linezolid treatment [43]. This indicates that the LVRE developed during linezolid treatment. Even though LVRE are rare, they are a concern since only daptomycin and tigecycline can be used for treatment of LVRE infections [44].

## Conclusion

The present study highlights the emergence and spread of seven VREfm and VVEfm clusters in Denmark. The spread of the VVE cluster, ST1421-CT1134 *vanA E. faecium*, in Denmark is of high concern, especially since VVE diagnostics are challenging. Therefore, this cluster is likely to be underdetected, which facilitates further spread. In 2015, the Danish national VRE programme produced WGS data on clinical VRE and VVE isolates. It is costly but allows for further characterisation of isolates for surveillance and outbreak detection and research without additional laboratory work. Currently, cgMLST is being used to follow the spread of VREfm and VVEfm both within the individual hospitals and between hospitals even outside the regions and too other countries. In addition, cgMLST is used for comparison for VRE isolates imported from abroad. We detected only a small proportion of LVRE (1.1%) our VREfm and VVEfm isolates. Nevertheless, this is of concern since the treatment options for LVRE are extremely limited. To reduce further development of LVRE, consumption of linezolid should be kept to a minimum.

## Supplementary Data

146000 Supplement
