# Supplementary material for: Surveillance of vancomycin-resistant enterococci reveals shift in dominating clusters from vanA to vanB Enterococcus faecium clusters, Denmark, 2015 to 2022
Source: Euro Surveill. 2024 Jun 6;29(23):2300633. doi: 10.2807/1560-7917.ES.2024.29.23.2300633 (PMC11158013; doi:10.2807/1560-7917.ES.2024.29.23.2300633)

This supplementary material is hosted by *Eurosurveillance* as supporting information alongside the article “*Surveillance of vancomycin-resistant enterococci reveals shift in dominating clusters from vanA to vanB Enterococcus faecium clusters, Denmark, 2015 to 2022*”, on behalf of the authors, who remain responsible for the accuracy and appropriateness of the content. The same standards for ethics, copyright, attributions and permissions as for the article apply. Supplements are not edited by *Eurosurveillance* and the journal is not responsible for the maintenance of any links or email addresses provided therein.

Figure S1. Incidence of VRE and VVE (Numbers/1000 bed-days) per region per year, Denmark 2015-2022

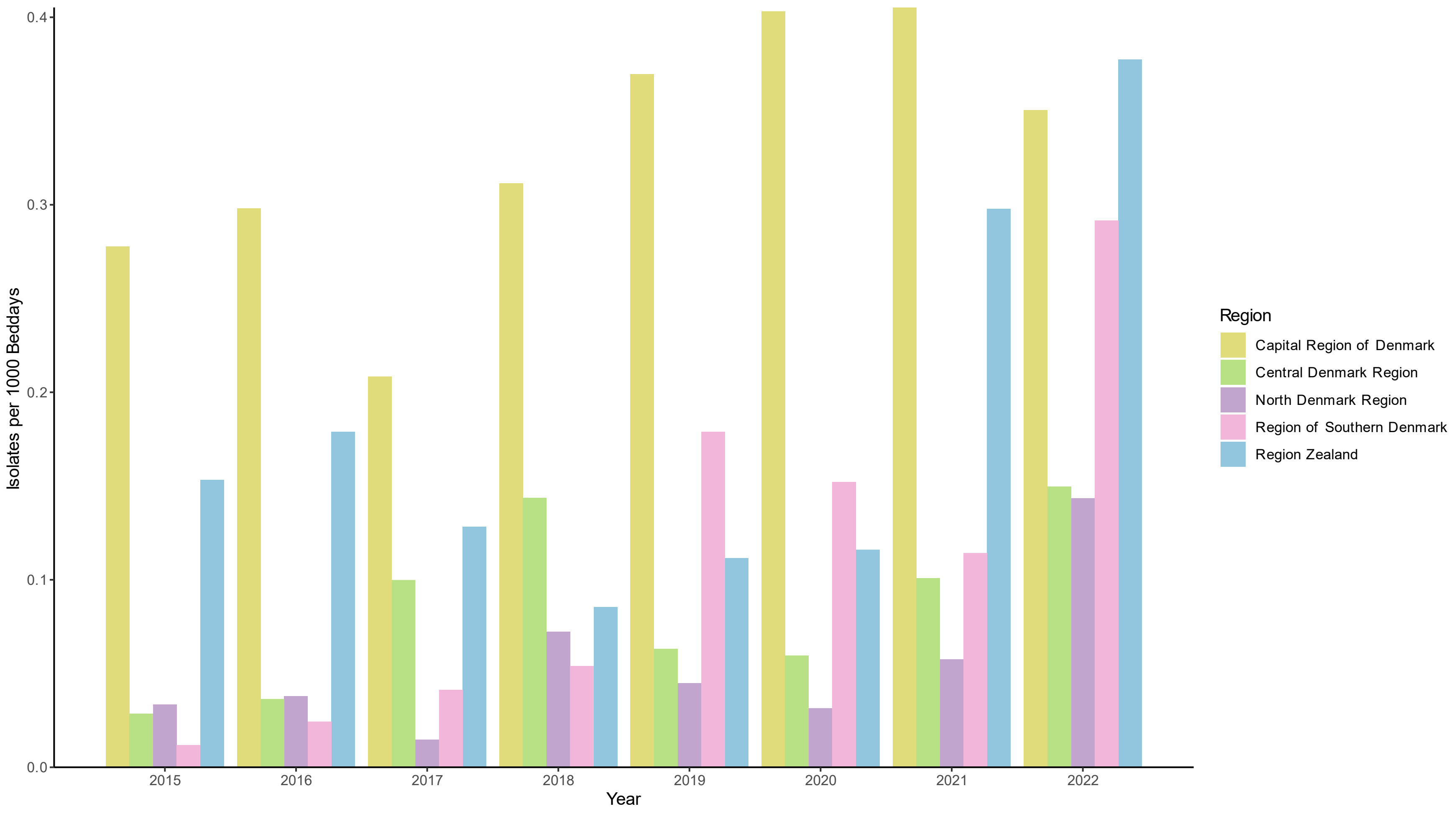

Supplement: Supplement [file 23-00633_HAMMERUM_SupplementaryFigureS1.pdf]
